# Regulation of Hepatic UGT2B15 by Methylation in Adults of Asian Descent

**DOI:** 10.3390/pharmaceutics10010006

**Published:** 2018-01-07

**Authors:** Steffen G. Oeser, Jon-Paul Bingham, Abby C. Collier

**Affiliations:** 1 Molecular Biosciences and Bioengineering, University of Hawaii, Manoa, 1955 East-West Rd. #218, Honolulu, HI 96822, USA; steffengo@gmail.com (S.G.O.); jbingham@hawaii.edu (J.-P.B.); 2 Faculty of Pharmaceutical Sciences, 2405 Wesbrook Mall, University of British Columbia, Vancouver, BC V6T1Z3, Canada

**Keywords:** glucuronidation, obesity, sex, polymorphisms

## Abstract

The hepatic uridine 5′-diphosphate-glucuronosyl transferases (UGTs) are critical for detoxifying endo- and xenobiotics. Since UGTs are also dynamically responsive to endogenous and exogenous stimuli, we examined whether epigenetic DNA methylation can regulate hepatic UGT expression and differential effects of ethnicity, obesity, and sex. The methylation status of UGT isoforms was determined with Illumina Methylation 450 BeadChip arrays, with genotyping confirmed by sequencing and gene expression confirmed with quantitative reverse transcriptase polymerase chain reaction (q-RT-PCR). The UGT1A3 mRNA was 2-fold higher in females than males (*p* < 0.05), while UGT1A1 and UGT2B7 mRNA were significantly higher in Pacific Islanders than Caucasians (both *p* < 0.05). Differential mRNA or methylation did not occur with obesity. The methylation of the UGT2B15 locus cg09189601 in Caucasians was significantly lower than the highly methylated locus in Asians (*p* < 0.001). Three intergenic loci between UGT2B15 and 2B17 (cg07973162, cg10632656, and cg07952421) showed higher rates of methylation in Caucasians than in Asians (*p* < 0.001). Levels of UGT2B15 and UGT2B17 mRNA were significantly lower in Asians than Caucasians (*p* = 0.01 and *p* < 0.001, respectively). Genotyping and sequencing indicated that only UGT2B15 is regulated by methylation, and low UGT2B17 mRNA is due to a deletion genotype common to Asians. Epigenetic regulation of UGT2B15 may predispose Asians to altered drug and hormone metabolism and begin to explain the increased risks for adverse drug reactions and some cancers in this population.

## 1. Introduction

The uridine 5′-diphosphate-glucuronosyl transferases (UGTs, E.C. 2.4.1.17) primarily eliminate xeno- and endobiotics in humans through conjugating them with a polar sugar [1]. Alone, or in conjunction with other enzymes, the UGTs are involved in the clearance of more than 90% of clinically relevant drugs from the body [2]. Based on nucleotide sequence analysis, 22 human UGT proteins have been identified in 4 families, with the UGT1A and UGT2B subfamilies being most critical for hepatic metabolism [3,4,5].

The UGT enzymes are genetically polymorphic, with over 200 variant alleles described for the UGT1 and UGT2 subfamilies that can influence enzymatic function, cellular trafficking, or gene expression, modifying individual drug and endobiotic exposure [6]. Additional variants have also been identified, but have not been characterized for function. Moreover, although some of these single nucleotide polymorphisms (SNPs) are functional, changes in total glucuronidation also tend to occur when many polymorphisms are inherited together as a haplotype [6].

In addition to genetic factors, UGT enzymes are known to be regulated by environmental, xenobiotic, and endobiotic exposure, but at present there is limited information regarding epigenetic regulation, such as the effects of methylation. Methylation of UGT1A1 has been associated with colon cancer and with altered drug disposition in cancer [7,8]. More recently, a link between DNA methylation and the regulation of UGT1A1 protein expression and activity in healthy human livers has been presented [9]. This latter study implies that epigenetics are not merely a disease-state effect in UGT disposition, but may be intimately involved in modifying basal UGT enzyme expression. Therefore, examining epigenetic modifications that alter mRNA levels of UGT enzymes in common demographics, such as sex, ethnicity, and obesity, may help determine how glucuronidation varies in mixed populations. This can help determine demographic contributions to the hepatic disposition of compounds.

If glucuronidation is dysregulated or differs inherently between members of mixed populations, it is important to understand the mechanisms for these changes. These mechanisms may present druggable targets in themselves for preventing or mitigating disease and could be exploited to prevent adverse drug reactions. Moreover, epigenetic mechanisms may provide insight into the differential susceptibility of some ethnicities to diseases and syndromes, including cancer. The purpose of this study was to determine the differential methylation of all hepatic UGT isoforms in a well-characterized cohort of human livers and investigate whether hepatic methylation of UGTs is associated with sex, ethnicity, or obesity.

## 2. Materials and Methods

### 2.1. Tissue Availability and Collection

Liver samples (*n* = 24) were collected from the Hawaii Human Biorepository, which collects samples with informed consent from non-surviving organ donors’ families, and with permission for future experimentation. The characteristics of this set of livers were as follows: Median age 52 ± 13 (range: 20–69), Males *n* = 15 and Females *n* = 9, Ethnicity: Caucasian *n* = 9 (38%), Asian *n* = 11 (33%), and Pacific Island (PI) *n* = 4 (16%). Three unknowns were excluded from analysis; moreover, one each of Caucasian and Asian were excluded from the methylation analysis due to poor sample quality. “Asian” in this study is reflective of Japanese, Chinese, or Korean ethnicity. The Body Mass Index (BMI) ranged from normal (20.8 kg/m^2^) to morbidly obese (52.4 kg/m^2^) with a median of 30.7 ± 8.5. For analysis, samples were grouped into two discrete categories, normal weight (NW) and overweight (OW), which corresponded to BMI ranges of 18.5–24.9 kg/m^2^ and above 25 kg/m^2^, respectively. These studies were approved by the Hawaii Institutional Review Board for Human Subjects (CHS #21144).

### 2.2. Liver Samples

Tissue samples (1 cm) were extracted from the anterior portion of the right lobe closest to the inferior vena cava from frozen livers. All livers were collected and archived (flash frozen) within 6 h of brain death, and these tissues were from livers that had never been thawed. None of the livers was diseased. Tissues samples were disrupted on liquid nitrogen using a mortar and pestle and stored at −80 °C until nucleic acid extraction.

### 2.3. DNA and RNA Extraction

DNA, RNA, and protein were extracted from 20 mg of disrupted liver sample according to the manufacturer’s instructions (Qiagen AllPrep DNA/RNA/Protein Mini kit, Valencia, CA, USA). Nucleic acid purity was examined using a Nanodrop spectrophotometer (ThermoFisher Scientific, Wilmington, DE, USA), and quality was examined with a Bioanalyzer (Agilent, Palo Alto, CA, USA). To further purify DNA, ethanol precipitation was performed and the pellet re-dissolved in nuclease free water.

### 2.4. DNA Methylation Analysis

The Illumina Infinium Methylation Assay (Illumina Inc., San Diego, CA, USA) was performed as per the manufacturer’s instructions. This assay covers 99% of RefSeq genes and 95% of Cytosine-guanine (CpG) islands in the human genome. The entire genome was interrogated and examined, but results with respect to UGT genes were the focus of this study. All CpG interrogation sites associated with all UGT genes were analyzed, but only significant results are presented. The DNA samples (1 μg) were first treated with sodium bisulfite using a Zymo Research Bisulfite conversion kit (Zymo Irvine, CA, USA) to deaminate un-methylated cytosine to produce uracil. Extraction, analysis, and visualization of the methylation data were performed using the Illumina Genome Studio software (version 2011.1) with methylation and genome viewer plug-ins (version 1.9.0, Illumina, San Diego, CA, USA). Differential methylation analysis was performed for grouped data utilizing the Illumina custom error model, resulting in a differential methylation score (diffscore) that provides directionality to the *p*-value. The diffscore was converted to an adjusted *p*-value with the formula: *p*-value = 1/((10^|DiffScore|^)/10).

### 2.5. Gene Expression via Quantitative Reverse Transcriptase Polymerase Chain Reaction (q-RT-PCR)

Total RNA (1 μg) was converted to cDNA using a qScript™ cDNA synthesis kit from Quanta Biosciences (Gaithersburg, MD, USA) according to the protocol provided. After mixing, reaction was incubated using the following conditions: 5 min at 25 °C, 30 min at 42 °C, 5 min at 85 °C, and then held at 4 °C for at least 5 min. From the resulting 20 μL reaction, 4 μL of cDNA product was then combined with forward and reverse qPCR primers (Table 1) to a final concentration of 500 nM. This reaction was then combined with an equal volume of 2× Roche Power SYBR Green Master Mix containing ROX. Samples were cycled on an Applied Biosystems 7900HT qPCR machine (ABI, Foster City, CA, USA) as follows: 2 min at 50 °C, 10 min at 95 °C, then 40 cycles of 15 s at 95 °C, and 1 min at 60 °C. Additionally, a dissociation step was performed at the end of the run, utilizing a +1 °C ramp rate per second from 60 °C to 95 °C with continuous detection, to ensure that only a single product was formed. Data for q-RT-PCR were displayed utilizing Applied Biosystems SDS software version 2.1 (ABI, Foster City, CA, USA). All mRNA expression C_T_ values were normalized against Beta-Glucuronidase (GUSB) before relative quantitation. Resulting C_T_ values were analyzed and converted to fold change differences using the ΔΔC_T_ method for relative quantitation [10].

### 2.6. Genotyping of the UGT2B17 Deletion

The presence or absence of the UGT2B17 gene deletion was determined. The C and J primer pairs (Table 2) were combined into a duplex reaction, generating a 316 bp fragment from within the UGT2B17 gene or an 884 bp fragment spanning the deletion breakpoint, respectively. Upon electrophoresis, individuals homozygous for the presence of UGT2B17 (Ins/Ins) show a single 316 bp product, individuals homozygous for the deletion show a single 884 bp product (Del/Del), and heterozygotes (Ins/Del) show both products with a reduction after the 316 bp fragment [11]. The PCR for the C and J primer pairs was carried out in 25 μL reactions containing 22.5 μL of Platinum Blue PCR Supermix (Invitrogen, Grand Island, NY, USA), 100 pmol each of forward and reverse primer, and 30 ng of genomic DNA. The cycle conditions were 94 °C for 3 min, 35 cycles of 94 °C for 30 s, 60 °C for 30 s, 72 °C for 3 min, and then finishing with a 4 °C hold.

### 2.7. UGT2B15 Polymerase Chain Reaction (PCR) Amplification for Promoter Region

The primers for the promoter region were designed utilizing Primer-BLAST software [12]. The A primer pair listed in Table 3 was used to amplify a 3368 bp region of the UGT2B15 promoter, and after ethanol precipitation to clean-up the PCR product, primer pair B was used to amplify that product to produce a final, specific, 3224 bp fragment for sequencing (Table 2). PCR for both reactions was carried out in a 10 μL reaction volume consisting of 9 μL of Platinum Blue PCR Supermix (Invitrogen, Grand Island, NY, USA), 1 pmol each of forward and reverse primer, and 20 ng of DNA. The cycle conditions were 94 °C for 3 min, 25 cycles of 94 °C for 30 s, 45 °C for 30 s, 72 °C for 3.5 min, and then finishing with a 4 °C hold. For SNP examination, samples were cleaned via ethanol precipitation and cycle sequenced utilizing the B forward PCR primer in addition to the sequencing primers for the promoter region listed in Table 3.

### 2.8. UGT2B15 PCR Amplification for 3′ Untranslated Regulatory Region

The primers for the untranslated regulatory region were designed utilizing the Primer-BLAST software [12]. The D primer pair listed in Table 3 was used to amplify 2567 bp of the UGT2B15 3′ untranslated regulatory region and after ethanol precipitation to clean-up the PCR product, primer pair E was used to amplify that product to produce a 2401 bp region for sequencing (Table 3). PCR for both reactions was carried out in a 10 μL reaction volume consisting of 9 μL of Platinum Blue PCR Supermix (Invitrogen, Grand Island, NY, USA), 1 pmol each of forward and reverse primer, and 20 ng of DNA. The cycle conditions were 94 °C for 3 min, 25 cycles of 94 °C for 30 s, 49 °C for 30 s, 72 °C for 3.5 min, and then finishing with a 4 °C hold. Samples were cleaned via ethanol precipitation and cycle sequenced utilizing the E forward PCR primer in addition to the listed sequencing primers for the untranslated regulatory region in Table 3. Products (300 ng) were electrophoresed on 2% (*w*/*v*) Tris-buffered EDTA (TBE) agarose gel and sized compared to a DNA ladder (Thermo Fisher, Wilmington, DE, USA) with imaging on a Typhoon Scanner 9410 (GE Healthcare, Chicago, IL, USA).

### 2.9. Cycle Sequencing

Each sample was sequenced in duplicate, combining 7.5 ng of PCR template with 2.5 pmol of sequencing primer in a total volume of 5 μL in a 96-well polypropylene half-skirted sequencing plate (Applied Biosystems, Foster City, CA, USA). Each 5 μL reaction was then mixed with 1.5 μL of nuclease free water, 1.5 μL of 5X Applied Biosystems Sequencing Buffer, and 2 μL of BigDye^®^ Terminator v3.1 Ready Reaction Enzyme Mix (Life Technologies, Grand Island, NY, USA). Samples were mixed, sealed, and sequenced under the following cycling conditions: 96 °C for 1 min followed by 25 cycles of 96 °C for 10 s, 50 °C for 5 s, and 60 °C for 4 min and terminating with a 4 °C hold until samples were ready for purification.

Samples were cleaned according to the BigDye^®^ Terminator v3.1 protocol and sequenced utilizing the Applied Biosystems DNA Analyzer 3730xl Sequencer (Foster City, CA, USA). Resulting chromatograms were examined via 4Peaks software v1.7.2 (Mek & Tosj, Amsterdam, The Netherlands), and sequences were extracted and examined for SNPs using Aliview to align and trim the sequences (Uppsala University, Uppsala, Sweden).

The resulting sequences were then entered into the BLAST program and aligned to the UGT2B15 gene (Genbank ID: NC_0000012.4). Sequences were examined and nucleotide calls recorded for each sample at each position where a known or suspected SNP exists (indicated in the BLAST program in the SNP annotation database).

### 2.10. Statistical Analyses

Statistical analysis was performed using the GraphPad Prism Program version 5.02 (Graph Pad, San Diego, CA, USA). Normality of the data was checked using D’Agostino–Pearson’s test and variance verified using the Bartlett’s test. A one-way analysis of variance (ANOVA) with Bonferroni’s post-hoc comparison was used if the data were found to be normally distributed; alternatively, the Kruskal–Wallis one-way ANOVA was performed with Dunn’s post hoc analysis. For binary data sets (sex), student’s *t*-tests were performed with α = 0.05.

## 3. Results

### 3.1. Summary of Methylation Beta Values for All UGT-Associated Loci

**Figure 1:**
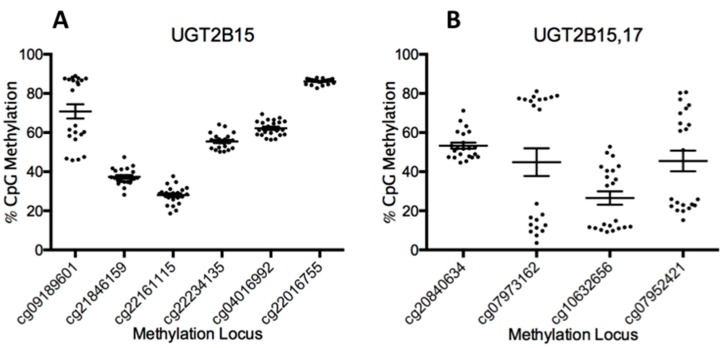
Grouped data showing the methylation beta values (*y*-axis) for each sample at a particular Cytosine-Guanine (CG) locus (*x*-axis) UGT2B15 (**A**) and UGT2B15/17 (**B**). Because UGT2B15/2B17 were formed by a gene duplication event, they also share common loci. Values above 70% are generally considered to be fully methylated on both strands and values below 30% are typically un-methylated on both strands with intermediate values indicating that only one of the two strands are methylated.

Inspection of the methylation status of all the UGT loci demonstrated that for UGT1A isoforms, most of the loci were generally moderately or hyper-methylated, but significant differential methylation was not occurring between samples. However, for UGT2B isoforms, at least four loci have differential methylation occurring with a clear splitting pattern between samples. These loci are associated with UGT2B15 (cg09189601) and both UGT2B15 and 2B17 (cg07973162, cg10632656, and cg07952421, Figure 1). No significant differences in methylation patterns were attributed to age, sex, or BMI for any of the four differentially methylated loci associated with UGT2B15 and UGT2B17.

**Figure 2:**
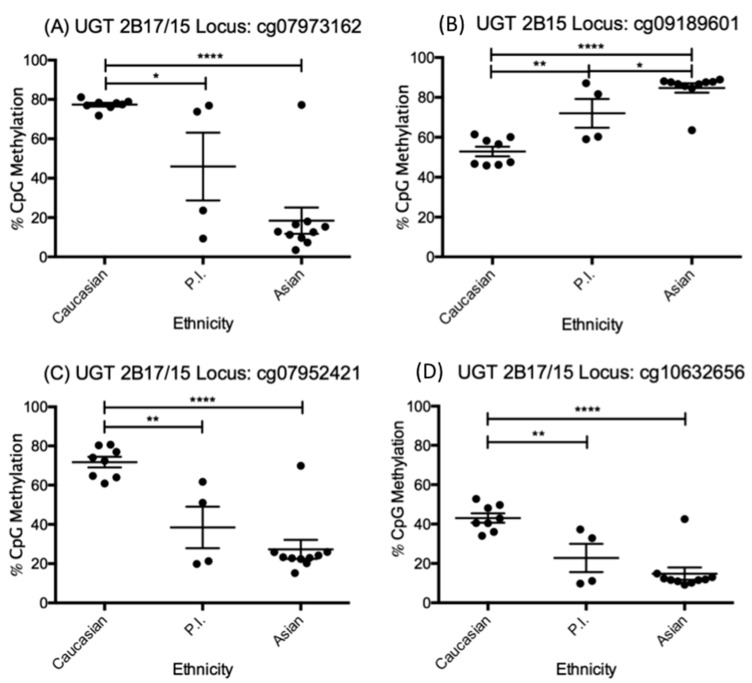
Graphs showing beta values (β) for differentially methylated loci associated with UGT2B15 and 2B17, grouped according to ethnicity with separate columns for Caucasians (*n* = 8), Pacific Islanders (PI, *n* = 4) and Asians (*n* = 10). The UGT isoform and the specific loci where the methylation is taking place are listed in the title for each individual graph such that loci cg07973162 (**A**), cg10632656 (**B**), cg07952421 (**C**), and cg09189601 (**D**) show different patterns of methylation between Asians, PI and Caucasians for UGT2B15 and UGT2B17. Statistical significance is listed for *p*-values ≤0.05 (*), ≤0.01 (**), and ≤0.0001 (****). As compared to PCR, two samples (one Caucasian and one Asian) were lost to analysis due to low sample quality.

The only difference attributed to differential methylation was ethnicity (Figure 2). For locus cg09189601, which is associated with UGT2B15 only, we observed hyper-methylation (90%) in Asians (*n* = 8) compared to the moderate methylation (50–60%) in Caucasians (*n* = 10, *p* < 0.0001), which would imply less transcription of UGT2B15 in Asians. In contrast, at loci cg07973162, cg10632656, and cg07952421 hepatic UGT2B15 and 2B17 were hypo-methylated in Asians (<20%) compared to the more highly methylated (40–80%) Caucasian samples (*p* < 0.0001 for all loci).

The Pacific Island cohort in our study (PI, *n* = 4) displayed two samples that reflected Asian methylation patterns and two samples that reflected Caucasian patterns. Due to a limited sample size and the split nature of their methylation pattern, no significant differences in methylation patterns were determined. The ethnic makeup of our PI group was ≥50% (i.e., at least one parent PI) to define ethnicity. However, for Asian and Caucasian populations 100% (i.e., both parents of this race) defined ethnicity, hence we cannot rule out admixed genetic information in PI from other ethnic groups, and this likely explains the splitting observed. Notably, intermarriage of PI/Caucasian and PI/Asian is common in Hawaii.

### 3.2. The mRNA Expression of UDP-Glucuronosyl Transferase (UGT) Isoforms in This Liver Cohort

To determine differences in UGT mRNA expression and confirm methylation effects, we performed q-RT-PCR on all hepatic UGT1A and 2B isoforms. Obesity was not associated with any significant differences in mRNA levels for any UGT1A or 2B isoforms. Only UGT1A3 showed significant differences in mRNA levels with sex, where females had 2-fold higher mRNA expression than males (*p* = 0.02, data not shown).

**Figure 3:**
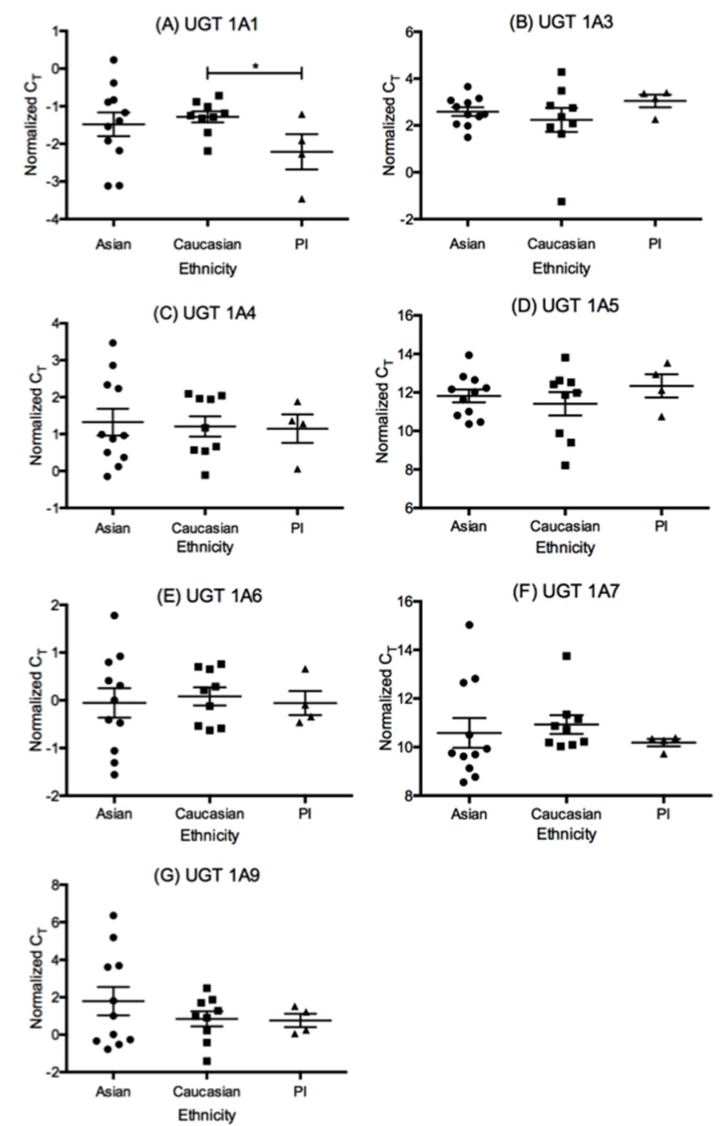
The quantitative PCR ΔΔC_T_ values in Asian (A, *n* = 11), Caucasian (C, *n* = 9), and Pacific Islander (PI, *n* = 4) ethnicities for all liver-specific UGT1A isoforms examined. Statistical significance is listed for *p*-values ≤0.05 (*) Note that higher ΔΔC_T_ means lower level of mRNA. (**A**–**G**) represent results for each individual UGT1A subfamily isoform.

Ethnicity was associated with significant differences in mRNA abundance for UGT1A1, where Caucasians had significantly lower mRNA levels than PI (higher ΔC_T_**,** *p* = 0.03, Figure 3). Caution should be exercised with these data, as the number of samples in the PI group is fairly low for statistical comparison.

**Figure 4:**
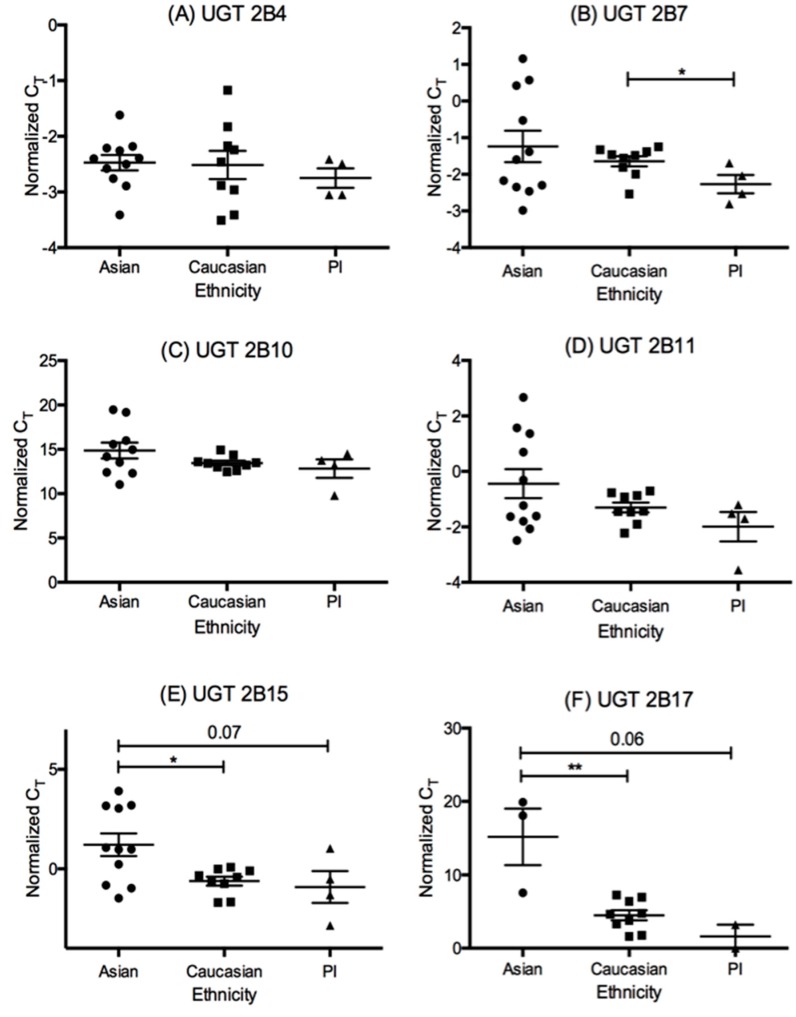
The quantitative PCR ΔC_T_ values in Asian (A, *n* = 11), Caucasian (C, *n* = 9), and Pacific Islander (PI, *n* = 4) ethnicities for all examined UGT2B isoforms in liver. Statistical significance is listed for *p*-values ≤0.05 (*), ≤0.01 (**), and *p* values approaching significance (<0.1). Note that a higher ΔC_T_ means a lower level of mRNA. (**A**–**F**) represent results for each individual UGT2B subfamily isoform.

Gene expression data for UGT2B samples demonstrated significantly lower UGT2B7 mRNA levels in Caucasians (higher ΔC_T_) as compared to PI (*p* = 0.038, Figure 4). When mRNA expression of UGT2B15 (Figure 4) was determined across the three ethnicities, Asians show significantly lower mRNA expression than Caucasians (*p* = 0.02) and approached significance for PI (*p* = 0.08). Finally, for UGT2B17 (Figure 4), Asians exhibited significantly lower mRNA levels than Caucasians (*p* = 0.001) and approached significance as compared to PI (*p* = 0.08). These data translate to a 3-fold significant downregulation in UGT2B15 in Asians as compared to Caucasians and PI. For UGT2B17, there is no fold change between Caucasians and PI, but there is a 1500-fold downregulation between Caucasians and the three Asian samples that produced gene expression data for this isoform.

### 3.3. UGT2B15 and UGT2B17 Genetic Analysis 

Comparison of SNP data to mRNA levels demonstrates that the decreased expression for UGT2B15 in these samples is not caused by any of the known UGT2B15 SNPs in the areas interrogated. Deletion genotyping of UGT2B17 demonstrated that eight of nine Asian samples and two of the PI samples have the UGT2B17 Del/Del genotype. Caucasian samples had at least one copy of the UGT2B17 gene, with three Ins/Ins, four Ins/Del, and two samples that failed genotyping. The two PI samples that did not have the deletion are of Ins/Del genotype. These genotyping results are consistent with the q-RT-PCR data for UGT2B17, where Asian samples had either an absent, or an extremely low, expression of UGT2B17. The deletion polymorphism is the major cause for this low UGT2B17 gene expression for the Asian samples and also explains the hypo-methylated CpG loci in the UGT2B15/2B17 intergenic region that reside within the deletion.

## 4. Discussion

The critical finding of this study is that the mRNA expression of hepatic UGT2B15 is partially regulated by methylation in people of Asian descent, causing decreased mRNA expression. Moreover, while UGT2B17 showed differential methylation in Asians, lower levels of mRNA are caused by a deletion genotype and not epigenetics. The novel finding from the UGT2B17 studies is that methylation of the three loci cg07973162, cg07952421, and cg10632656 in the Illumina platform is predictive of the deletion genotype. This is because the range targeted by the Illumina methylation assay covers the deletion, as confirmed by genotyping. Other than the findings presented for individuals of Asian descent, methylation did not regulate UGT1A or UGT2B isoforms with age, ethnicity, obesity, or sex in this cohort. Finally, although not regulated by methylation, hepatic UGT1A3 mRNA levels were higher in females than males, while UGT1A1 mRNA levels were significantly higher in PI than Caucasians.

Because females showed significantly higher mRNA expression of UGT1A3 compared to males, this may have implications for sex-differences in drug and endobiotic metabolism and disposition if it is indicative of protein levels. The UGT1A3 isoform acts on some drugs and is an important metabolic pathway for bile acids [13]. Similarly, PI had higher mRNA expression of UGT1A1 than Caucasians. This is one of the most active UGTs in the liver, with wide substrate affinity. Differences in UGT expression between these two ethnic groups may begin to explain some of the differences in drug and chemical response in an admixed population. There are no reports in the literature indicating disproportionate glucuronidation in PIs, but, rather than contradicting our data, this is due to a lack of study in this ethnic population. A recent study by our laboratory has demonstrated that morbid obesity in pregnancy is associated with higher levels of unconjugated bilirubin in the blood of obese PI women and in their neonates (unpublished data). This suggests that there may be ethnic-specific effects on UGT in the PI population and is deserving of further study. While we did not find any associations with obesity in the current study, it has recently been reported that UGT2B17 can vary with obesity but only in males [14]. Given the mixed male and female cohort used, as well as the deletion of UGT2B17 in approximately one third of our samples, we would not have been empowered to confirm this finding.

For the locus associated solely with UGT2B15, Asian samples were hyper-methylated (90%) compared to the moderate methylation (50–60%) in Caucasians. This is opposite to the three common UGT2B15/2B17 loci, which are hypo-methylated in Asians (<20%) compared to the more highly methylated (40–80%) Caucasian samples. Taken together, these results imply a regulatory mechanism by which both UGT2B15 and 2B17 are methylated simultaneously at the intergenic region and UGT2B15 is modified at another site to modulate its glucuronidation separately. Because these two isoforms are the result of a gene duplication event in the recent past, this complex differential regulation is empirically sensible. The mRNA expression patterns are not concordant with methylation data that is typically observed in connection with gene promoters, which was expected because methylation occurs within intergenic areas for UGT2B15 and UGT2B17. In general, regulatory methylation occurs at hypo-methylated sites commonly located in CpG islands within 1.5 kb of the transcription start site of a gene, while hyper-methylated sites tend to be located in the distal intergenic and gene body regions [15,16,17]. Another potential explanation for this is that the liver is highly enriched in 5 hmC and the bisulfite technique does not discriminate between 5 mC and 5 hmC. Since these two CpG modifications have opposing effects, this may also explain why mRNA levels are not concordant with traditional methylation patterns. Finally, methylation patterns presenting with a beta value of 50% could represent an admixed cell population where half the cells are methylated and half are not. This is highly unlikely to be the case in our study, since the cells in the small 1–2 g pieces of human liver would be exposed to the same endo- and xenobiotic stimuli and concordance between a majority of the UGT-related CpG sites was high.

In addition to epigenetic mechanisms, functional and clinically relevant polymorphisms in UGT2B15 and 2B17 that affect drug and chemical disposition exist. This includes the UGT2B15*2 polymorphism, where reduced lorazepam and S-oxazepam clearance occurs [18,19], as well as the UGT2B17*2 and UGT2B17 deletion genotypes, which both reduce MK-7246 and exemestane clearances, potentially leading to toxic accumulation [20,21]. Polymorphisms may also increase activity, such as with UGT2B15*4, which has a C > A nucleotide change in the coding region, increasing UGT2B15 catalysis [18,19]. Polymorphisms in UGT2B15 and 2B17 have been implicated in modifying the clearance of a wide range of drugs, reviewed in: [22]. Here, an analogy can be made with methylation because if Asians have differential methylation patterns compared to Caucasians for UGT2B15, they may also have altered glucuronidation capacity.

In addition to drugs and chemicals, UGT2B15 and 2B17 have been investigated in relation to risks for cancer and other endocrine diseases [23]. This is especially true with regard to the UGT2B17 deletion phenotype, which is more prevalent in the Asian population [24] and is particularly important because UGT2B17 is more active than UGT2B15 for testosterone glucuronidation [25,26]. Polymorphisms in both of these genes have been shown to alter steroid metabolism and also are predictors of fat mass in men [27]. Most studies indicate that genetic polymorphisms affecting UGT2B15 and/or 2B17 are risk factors for prostate cancer [28,29,30,31]. Although lifestyle and environmental factors account for some prostate cancer in Asians, these only account for a small proportion of the relative risk [32]. These prior research efforts have elucidated many of the biological effects of UGT2B15 and UGT2B17 polymorphisms, but the impact of epigenetic methylation has not previously been established. Our study implies that hyper-methylation of UGT2B15 in Asians may affect androgen disposition as compared to the Caucasian population and is a promising avenue for investigating some of the altered risk for androgen-related cancers in these populations.

Although this study contained only a relatively small sample size, making interactions between environment, genotype, and demographics difficult to establish, the numbers of samples for ethnicities, sex, and obesity were well-balanced. This is bolstered by our findings from the samples of PI ethnicity, where UGT genotypes showed commonality with Asians for two of the samples and for Caucasians with the other two samples. Since most of the PI participants were of mixed heritage (≥50% PI, admixed with either Asian or Caucasian), these findings fit the rules of heritability. Moreover, a strong Asian linkage is not surprising, as PI are theorized to have originated in Asia and mixed extensively with Melanesians both depositing and accumulating genes into their genomes during colonization of the Pacific Islands [33], which has been further corroborated by both Y chromosome and mitochondrial DNA analyses [34]. Finally, our work is complemented by recent data demonstrating that hepatic UGT1A1 can be regulated during early human development by histone modification [35], a novel and interesting finding that supports the concept that UGTs can be regulated at the transcriptional level through genetic mechanisms. Having said this, we believe that future work in this area will need to encompass protein-level studies with Western blot of UGT2B15 and/or activity studies to confirm if these results found at the mRNA levels also follow through to the levels of enzyme proteins and their function.

A secondary finding here is that deletion of UGT2B17 is entirely predicted by methylation status, allowing for genotyping of the (Del/Del) allele via a methylation assay when all three UGT2B15/2B17 loci (cg07973162, cg07952421, and cg10632656) are hypo-methylated (<20%) due to the deletion of UGT2B17. In these samples, this occurred entirely within the Asian population of samples, which agrees with published research showing that this deletion is prevalent in Asian populations [24]. The deletion was also mirrored perfectly by the elimination of mRNA expression for UGT2B17. While it is currently very expensive to use methylation technologies, as the cost progressively reduces the validation of this finding may present an alternative avenue to sequencing or genotyping for determining the UGT2B17 deletion genotype.

## 5. Conclusions

In summary, methylation may be a mode of regulatory control for UGT2B15 in Asians, which likely acts in conjunction with SNP modification, reducing the enzyme’s expression. Impairment of UGT2B15 is of clinical significance with respect to risks for prostate cancer [29] and renal disorders [36] as well as drug metabolism. As this is a small sample size, a larger cohort will be needed to confirm these findings, although with a signal-to-noise ratio as high as we observed we expect the findings to be reproducible. Elucidating the genetic and environmental regulation of UGT2B15 and 2B17 can be useful for predicting susceptibility to cancer and adverse drug reactions, but more work on the precise mechanisms of UGT2B15 methylation, the interplay of methylation and SNPs, as well as the effects on drug and hormone handling in vivo are needed to tease out the clinical significance of these findings.

## Figures and Tables

**Table 1:** Uridine 5′-diphosphate-glucuronosyl transferases (UGT) primer sequences used to produce quantitative polymerase chain reaction (qPCR) results for gene expression analysis. The isoform examined, forward and reverse primer sequences, as well as publication source (reference number) are listed. Primers were from references. GUSB: Beta-Glucuronidase.

UGT	Forward Primer Sequence (5′-3′)	Reverse Primer Sequence (5′-3′)
1A1	AATAAAAAAGGACTCTGCTATGCT	ACATCAAAGCTGCTTTCTGC
1A3	TGTTGAACAATATGTCTTTGGTCTA	ACCACATCAAAGGAAGTAGCA
1A4	GAACAATGTATCTTTGGCCC	ACCACATCAAAGGAAGTAGCA
1A6	CATGATTGTTATTGGCCTGTAC	TCTGTGAAAAGAGCATCAAACT
1A8	GAAAGCACAAGTACGAAGTTTG	GGGAGGGAGAAATATTTGGC
1A9	TGGAAAGCACAAGTACGAAGTATATA	GGGAGGGAGAAATATTTGGC
1A10	GAAAGCACAGGCACAAAGTATA	GGGAGGGAGAAATATTTAGCAAC
2B4	TCTTTCGATCCAACAGCC	CATCTCTTAACCGCTGCTTGATA
2B7	GGAGAATTTCTCATGCAACAGA	CAGAACTTTCTATTATGTCACCAAATATTG
2B11	AGTAACATGACAGCAGAAAGGGCCAAT	AGACCTAAGGCATCTGGTTTATTCCCG
2B15	CTTCTGAAAATCTCGATAGATGGAT	CATCTTTACAGACTTGTTACTGTAGTCAT
2B17	TTTATGAAAAGTTCGATAGATGGAC	CATCTTCACAGACTTTATATTATAGTCAG
GUSB	AGCCAGTTCCTCATCAATGG	GGTAGTGGCTGGTACGGAAA

**Table 2:** Primer sequences for PCR reactions to characterize single nucleotide polymorphisms (SNPs) and deletions. The UGT isoform being examined, region of amplification, primer name, direction, and sequence as well as the resulting amplicon size are shown. UTR: untranslated region.

UGT	Region	Primer	Orientation	Sequence (5′-3′)	Amplicon (bp)
2B17	Gene	C	Forward	CCTGGAAGAGCTTGTTCAGA	316
2B17	Gene	C	Reverse	CTGCATCTTCACAGAGCTTT	316
2B17	Deletion	J	Forward	TGCACAGAGTTAAGAAATGGAGAGATGTG	884
2B17	Deletion	J	Reverse	GATCATCCTATATCCTGACAGAATTCTTTTG	884
2B15	Promoter	A	Forward	GGTCCCACTTCTTCAGATCAT	3368
2B15	Promoter	A	Reverse	GAGAGAAGGAAGAAGCCAGAAG	3368
2B15	Promoter	B	Forward	ACATAGGAAGGAGGGAACAGA	3224
2B15	Promoter	B	Reverse	TTCCTGCTGAGGGTTTGAAG	3224
2B15	UTR	D	Forward	TGGTGTGGATGTCCTTTCTG	2567
2B15	UTR	D	Reverse	GGCAGGAGAATGACTTGACTAC	2567
2B15	UTR	E	Forward	CTGCAGGTCTGTTGGAATTTG	2401
2B15	UTR	E	Reverse	GCAGTTGTAGTCCTAGCTTCTC	2401

**Table 3:** Sequencing primers used to obtain the sequence data for the PCR reactions. The UGT isoform being examined, region of amplification, primer name, binding DNA strand, and sequence are shown.

UGT Isoform	Region	Primer #	Strand	Sequence (5′-3′)
2B17	Gene	1	−	CTGGTCCCACTTCTTCAGAT
2B15	Promoter	2	+	GTTTGCAGATTTTTAATGAGGCA
2B15	Promoter	3	+	CTCCTAGGATTTGGCACCAG
2B15	Promoter	4	+	TTCTCTAATTTGACTCAGCTTCACA
2B15	Promoter	5	−	CTCAGCCCACCTGCAACC
2B15	Promoter	6	−	CCCCCTCTCCAGAATACACA
2B15	Promoter	7	−	TATCGTGGTGCAAGTAATGTCTTC
2B15	Promoter	8	−	TTATCCAATGGCTGTATTCTGTG
2B15	Promoter	9	+	ACTTTCCCACCGAAAATTCC
2B15	Promoter	10	+	TGCGTGGCAACTGTGATATT
2B15	Promoter	11	−	CAGGAAAAAGGAAATCCTCCA
2B15	Promoter	12	−	CTTTCGTGTGTAACTTTTGGATT
2B15	UTR	13	+	GAGGTTACTGCTGTCTCTTTGT
2B15	UTR	14	+	TGGTGTGGATGTCCTTTCTG
2B15	UTR	15	−	CCCTGGATCGAGCAGTCTTC
2B15	UTR	16	−	GACCAACCAATGAAGCCCCT
